# Bis(chlorido)(dimethyl­sulfoxide-κO)barium(II)

**DOI:** 10.1107/S160053681204069X

**Published:** 2012-10-03

**Authors:** Fabienne Gschwind, Martin Jansen

**Affiliations:** a Max Planck Institute for Solid State Research, Heisenbergstrasse 1, 70569 Stuttgart, Germany

## Abstract

The title compound, [BaCl_2_(C_2_H_6_SO)], forms a Ba_6_Cl_9_ cluster in which the BaCl_2_ units are connected *via* dimethyl­sulfoxide (DMSO) and chloride bridges. The central Cl atom of the Ba_6_Cl_9_ cluster is located on a threefold inversion axis and is coordinated octa­hedrally to six barium cations. In the crystal, the clusters are arranged in rows, which are inter­connected by the DMSO mol­ecules, forming a three-dimensional network.

## Related literature

For general background to barium complexes with chloride bridges, see: Yang *et al.* (2006 ▶); Arion *et al.* (2001 ▶); Fenske *et al.* (1993 ▶). For further information on chelated barium clusters with a central chloride atom, see: Drozdov *et al.* (1994 ▶). For examples of barium–DMSO complexes, see: Harrowfield *et al.* (2004 ▶); Pi *et al.* (2009 ▶). For a description of the Cambridge Structural Database, see: Allen (2002 ▶).
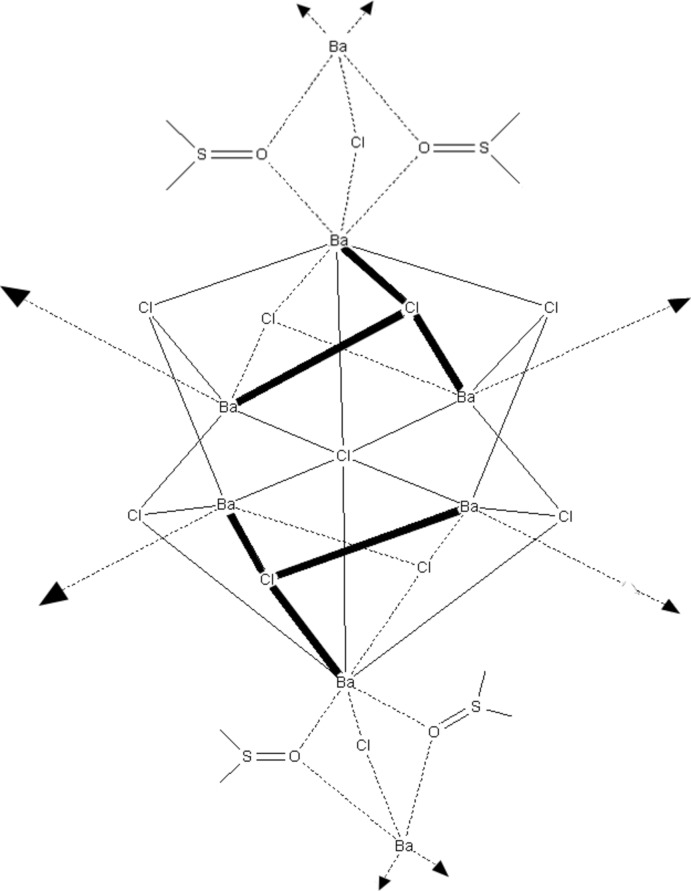


## Experimental

### 

#### Crystal data

[BaCl_2_(C_2_H_6_OS)]
*M* *_r_* = 286.37
Trigonal, 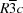

*a* = 15.680 (7) Å
*c* = 33.848 (6) Å
*V* = 7207 (5) Å^3^
*Z* = 36
Mo *K*α radiation
μ = 5.79 mm^−1^
*T* = 298 K
0.18 × 0.12 × 0.10 mm

#### Data collection

Stoe IPDS 2 diffractometer
Absorption correction: numerical (*X-SHAPE*; Stoe & Cie, 2009 ▶) *T*_min_ = 0.422, *T*_max_ = 0.595
28344 measured reflections
1807 independent reflections
1783 reflections with *I* > 2σ(*I*)
*R*_int_ = 0.059

#### Refinement

*R*[*F* ^2^ > 2σ(*F* ^2^)] = 0.023
*wR*(*F* ^2^) = 0.057
*S* = 1.25
1807 reflections
55 parameters
H-atom parameters constrained
Δρ_max_ = 0.51 e Å^−3^
Δρ_min_ = −0.54 e Å^−3^

### 

Data collection: *X-AREA* (Stoe & Cie, 2009 ▶); cell refinement: *X-AREA*; data reduction: *X-RED32* (Stoe & Cie, 2009 ▶); program(s) used to solve structure: *SHELXS97* (Sheldrick, 2008 ▶); program(s) used to refine structure: *SHELXL97* (Sheldrick, 2008 ▶); molecular graphics: *DIAMOND* (Brandenburg, 2006 ▶); software used to prepare material for publication: *publCIF* (Westrip, 2010 ▶).

## Supplementary Material

Crystal structure: contains datablock(s) I, global. DOI: 10.1107/S160053681204069X/hg5254sup1.cif

Structure factors: contains datablock(s) I. DOI: 10.1107/S160053681204069X/hg5254Isup2.hkl

Additional supplementary materials:  crystallographic information; 3D view; checkCIF report

## Figures and Tables

**Fig. 1.**
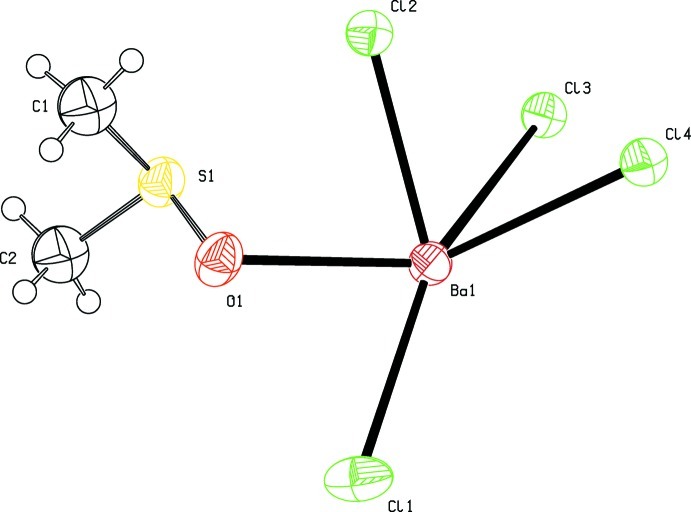
A view of the asymmetric unit of the title compound. Three of the four Cl atoms are located on special positions and have partial occupancies of Cl1 1/2, Cl3 1/6 and Cl4 1/3.

**Fig. 2.**
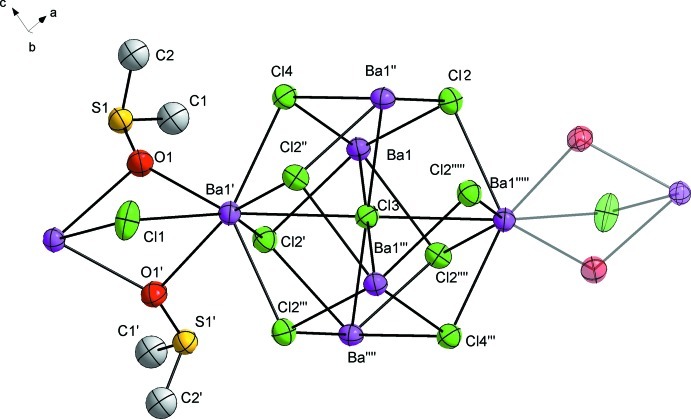
A view of the molecular structure of the title compound, with the atom numbering. The displacement ellipsoids are drawn at the 50% probability level. Symmetry codes: (1) -y, x - y, z (2) -x + y, -y, z (3) -x, -y, -z (4) x - y, x, -z (5) y, x + y, -z.

**Fig. 3.**
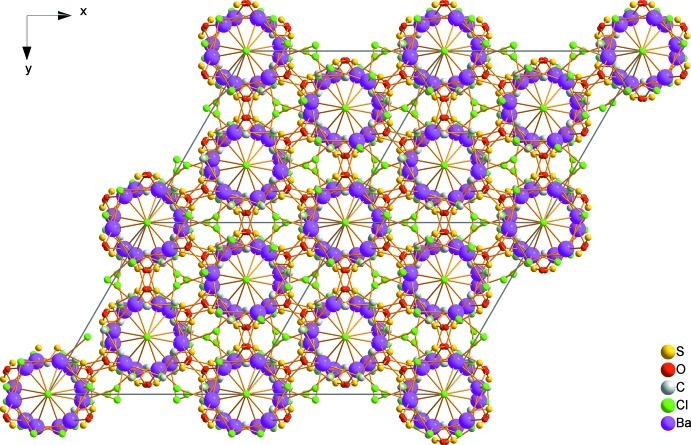
A view along the z-axis of the crystal packing of the title compound.

## supplementary crystallographic information

### Comment

The title compound crystallizes in the trigonal space group R3c and its asymmetric unit consists of one barium ion and four chloride ions (three of which are located on special positions and have partial occupancies: Cl1 1/2; Cl3 1/6; Cl4 1/3) and one DMSO solvent molecule (Fig. 1). The complete structure forms a Ba_6_Cl_9_ cluster (Fig. 2). Atom Cl3 occupies the center of the polyhedron located at position (0,0,0: 3); it is coordinated to six barium ions and has an octahedral configuration. Each barium ion sits on a corner of the cluster and coordinates *via* two O atoms of the DMSO molecule (O1 and its symmetry equivalent O1^i^; Ba1—O1 2.752 (3) Å, Ba1—O1^i^ 2.830 (1) Å; symmetry code: (i) *x* - *y* + 1/3, -*y* + 2/3, -*z* + 1/6) and one chloride (Ba1—Cl1 = Cl1—Ba1^i^ = 3.088 (1) Å) to the next BaCl cluster. The average Ba—O bond distance lies in the typical range for a Ba—O(DMSO) bond length (2.637–2.875 Å). The Ba—Cl bond distances in the title compound vary between 3.0888 (16)–3.3231 (11) Å, while a similar bridging Ba—Cl—Ba structure shows bond lengths between (3.114–3.253 Å).

The average Ba···Ba distance in the cluster is about 4.69 Å, while the distance between the two bridged barium ions is shorter at 4.3106 (19) Å.

Due to the high symmetry the Ba–DMSO bridge spreads out in all three dimensions (Fig. 3). In the *z*-dimension wheel-shaped structures of the rows of BaCl clusters are visible. The `DMSO-chloride' bridges are arranged around the wheels. The closest distance from the BaCl clusters is about 10.6 Å (measured between Cl3 and Cl3^ii^; symmetry code: (ii) 1/3 + *y*, 2/3 + *x*, 1/6 - *z*). There are no classical hydrogen bonds present but there is a small solvent accessible void of *ca* 63 Å^3^.

A literature search (Allen, 2002) revealed no similar barium-chloride clusters, but there are several examples of barium chloride bridged structures. For instance barium sulfonate complexes with layered structures (Yang *et al.*, 2006) or chloride bridged macrocyclic barium complexes (Arion *et al.*, 2001; Fenske *et al.* 1993). There exist also clusters of barium and O atoms with a bridging central chloride ion (Drozdov *et al.*, 1994). Furthermore, there exist different examples of barium DMSO complexes (Harrowfield *et al.*, 2004; Pi *et al.* 2009).

### Experimental

The title compound was obtained incidentally as a side-product in the following reaction:

Solution A: To a solution of BaCl_2_ (1 g) dissolved in methanol (10 ml) was added 1,5 g of tetraethylene glycol. Product B: Phosphomolybdic acid hydrate (0.25 g, 0.54 mmol) was dissolved in acetone (5 ml) and precipitated with an excess of cobaltocenium hexafluorophosphate (0.2 g) in acetonitrile (5 ml).

Product B was then dissolved in acetonitrile (10 ml) and precipitated with solution A. The precipitate was dissolved in hot DMSO (15 ml). After cooling the solution was layered with diethylether. A few colorless crystals the title compound appeared as a side-product after a few weeks.

### Refinement

Atoms C1 and C2 were treated isotropically due to thermal disorder. The H atoms were included in calculated positions and treated as rding atoms: C—H = 0.96 Å with *U*_iso_(H) = 1.5*U*_eq_(C). Potential Solvent Area Volume = 63.2 Å^3^. A small void of less than 1% was found in the crystal structure. It was not considered in the refinement.

### Crystal data

**Table:**

[BaCl_2_(C_2_H_6_OS)]	*D*_x_ = 2.375 Mg m^−^^3^
*M**_r_* = 286.37	Mo *K*α radiation, λ = 0.71073 Å
Trigonal, *R*3*c*	Cell parameters from 28704 reflections
Hall symbol: -R 3 2"c	θ = 1.5–57.3°
*a* = 15.680 (7) Å	µ = 5.79 mm^−^^1^
*c* = 33.848 (6) Å	*T* = 298 K
*V* = 7207 (5) Å^3^	Bloc, colourless
*Z* = 36	0.18 × 0.12 × 0.10 mm
*F*(000) = 4752	

### Data collection

**Table:**

Stoe IPDS 2 diffractometer	1807 independent reflections
Radiation source: fine-focus sealed tube	1783 reflections with *I* > 2σ(*I*)
Graphite monochromator	*R*_int_ = 0.059
Detector resolution: 6.67 pixels mm^-1^	θ_max_ = 27.3°, θ_min_ = 2.6°
ω and φ scans	*h* = −20→19
Absorption correction: numerical (*X-SHAPE*; Stoe & Cie, 2009)	*k* = −20→20
*T*_min_ = 0.422, *T*_max_ = 0.595	*l* = −43→43
28344 measured reflections	

### Refinement

**Table:**

Refinement on *F*^2^	Primary atom site location: structure-invariant direct methods
Least-squares matrix: full	Secondary atom site location: difference Fourier map
*R*[*F*^2^ > 2σ(*F*^2^)] = 0.023	Hydrogen site location: inferred from neighbouring sites
*wR*(*F*^2^) = 0.057	H-atom parameters constrained
*S* = 1.25	*w* = 1/[σ^2^(*F*_o_^2^) + (0.0207*P*)^2^ + 30.0844*P*] where *P* = (*F*_o_^2^ + 2*F*_c_^2^)/3
1807 reflections	(Δ/σ)_max_ < 0.001
55 parameters	Δρ_max_ = 0.51 e Å^−^^3^
0 restraints	Δρ_min_ = −0.54 e Å^−^^3^

### Special details

**Table:**

Geometry. All e.s.d.'s (except the e.s.d. in the dihedral angle between two l.s. planes) are estimated using the full covariance matrix. The cell e.s.d.'s are taken into account individually in the estimation of e.s.d.'s in distances, angles and torsion angles; correlations between e.s.d.'s in cell parameters are only used when they are defined by crystal symmetry. An approximate (isotropic) treatment of cell e.s.d.'s is used for estimating e.s.d.'s involving l.s. planes.
Refinement. Refinement of *F*^2^ against ALL reflections. The weighted *R*-factor *wR* and goodness of fit *S* are based on *F*^2^, conventional *R*-factors *R* are based on *F*, with *F* set to zero for negative *F*^2^. The threshold expression of *F*^2^ > σ(*F*^2^) is used only for calculating *R*-factors(gt) *etc*. and is not relevant to the choice of reflections for refinement. *R*-factors based on *F*^2^ are statistically about twice as large as those based on *F*, and *R*-factors based on ALL data will be even larger.

### Fractional atomic coordinates and isotropic or equivalent isotropic displacement parameters (Å^2^)

**Table:**

	*x*	*y*	*z*	*U*_iso_*/*U*_eq_	
S1	0.24622 (7)	0.39751 (7)	0.00510 (3)	0.0417 (2)	
O1	0.1864 (2)	0.38899 (19)	0.04210 (7)	0.0463 (6)	
C2	0.2400 (4)	0.4882 (3)	−0.02432 (13)	0.0576 (10)*	
H2A	0.1744	0.4620	−0.0344	0.086*	
H2B	0.2855	0.5063	−0.0459	0.086*	
H2C	0.2567	0.5452	−0.0085	0.086*	
C1	0.3718 (4)	0.4668 (4)	0.01944 (14)	0.0614 (11)*	
H1A	0.3880	0.4270	0.0359	0.092*	
H1B	0.3826	0.5241	0.0339	0.092*	
H1C	0.4128	0.4868	−0.0037	0.092*	
Cl1	−0.03305 (8)	0.3333	0.0833	0.0548 (4)	
Cl2	0.23027 (6)	0.18744 (6)	0.03813 (3)	0.03946 (18)	
Cl4	0.0000	0.0000	0.10413 (4)	0.0396 (3)	
Ba1	0.037103 (14)	0.191429 (13)	0.054807 (5)	0.03329 (8)	
Cl3	0.0000	0.0000	0.0000	0.0356 (4)	

### Atomic displacement parameters (Å^2^)

**Table:**

	*U*^11^	*U*^22^	*U*^33^	*U*^12^	*U*^13^	*U*^23^
S1	0.0425 (4)	0.0386 (4)	0.0401 (4)	0.0172 (4)	0.0055 (3)	−0.0018 (3)
O1	0.0454 (14)	0.0441 (14)	0.0408 (13)	0.0160 (12)	0.0088 (11)	−0.0018 (11)
Cl1	0.0436 (4)	0.0669 (9)	0.0617 (8)	0.0334 (4)	−0.0147 (3)	−0.0295 (7)
Cl2	0.0385 (4)	0.0382 (4)	0.0424 (4)	0.0197 (3)	0.0019 (3)	−0.0004 (3)
Cl4	0.0412 (4)	0.0412 (4)	0.0363 (7)	0.0206 (2)	0.000	0.000
Ba1	0.03337 (11)	0.03172 (11)	0.03353 (12)	0.01533 (8)	0.00041 (7)	−0.00300 (7)
Cl3	0.0367 (5)	0.0367 (5)	0.0334 (9)	0.0183 (3)	0.000	0.000

### Geometric parameters (Å, º)

**Table:**

S1—O1	1.530 (3)	Cl4—Ba1^iv^	3.2232 (13)
S1—C1	1.776 (5)	Cl4—Ba1^ii^	3.2232 (13)
S1—C2	1.778 (5)	Cl4—Ba1	3.2232 (13)
O1—Ba1^i^	2.752 (2)	Ba1—O1^i^	2.752 (2)
O1—Ba1	2.830 (3)	Ba1—Cl2^iv^	3.1528 (16)
C2—H2A	0.9600	Ba1—Cl2^v^	3.1968 (11)
C2—H2B	0.9600	Ba1—Cl3	3.3231 (11)
C2—H2C	0.9600	Ba1—Ba1^i^	4.3106 (16)
C1—H1A	0.9600	Ba1—Ba1^v^	4.6225 (10)
C1—H1B	0.9600	Ba1—Ba1^iii^	4.6225 (10)
C1—H1C	0.9600	Ba1—Ba1^iv^	4.776 (2)
Cl1—Ba1	3.0888 (16)	Cl3—Ba1^v^	3.3231 (11)
Cl1—Ba1^i^	3.0888 (16)	Cl3—Ba1^vi^	3.3231 (11)
Cl2—Ba1	3.1123 (16)	Cl3—Ba1^iii^	3.3231 (11)
Cl2—Ba1^ii^	3.1528 (16)	Cl3—Ba1^iv^	3.3231 (11)
Cl2—Ba1^iii^	3.1968 (11)	Cl3—Ba1^ii^	3.3231 (11)
			
O1—S1—C1	105.9 (2)	O1^i^—Ba1—Ba1^i^	40.11 (5)
O1—S1—C2	104.60 (19)	O1—Ba1—Ba1^i^	38.79 (5)
C1—S1—C2	98.7 (2)	Cl1—Ba1—Ba1^i^	45.751 (18)
S1—O1—Ba1^i^	146.52 (15)	Cl2—Ba1—Ba1^i^	95.556 (16)
S1—O1—Ba1	110.82 (13)	Cl2^iv^—Ba1—Ba1^i^	130.725 (16)
Ba1^i^—O1—Ba1	101.09 (8)	Cl2^v^—Ba1—Ba1^i^	107.06 (2)
S1—C2—H2A	109.5	Cl4—Ba1—Ba1^i^	119.27 (3)
S1—C2—H2B	109.5	Cl3—Ba1—Ba1^i^	161.700 (5)
H2A—C2—H2B	109.5	O1^i^—Ba1—Ba1^v^	173.27 (6)
S1—C2—H2C	109.5	O1—Ba1—Ba1^v^	114.60 (5)
H2A—C2—H2C	109.5	Cl1—Ba1—Ba1^v^	104.838 (18)
H2B—C2—H2C	109.5	Cl2—Ba1—Ba1^v^	103.16 (2)
S1—C1—H1A	109.5	Cl2^iv^—Ba1—Ba1^v^	43.656 (18)
S1—C1—H1B	109.5	Cl2^v^—Ba1—Ba1^v^	42.18 (2)
H1A—C1—H1B	109.5	Cl4—Ba1—Ba1^v^	99.24 (3)
S1—C1—H1C	109.5	Cl3—Ba1—Ba1^v^	45.933 (9)
H1A—C1—H1C	109.5	Ba1^i^—Ba1—Ba1^v^	139.897 (6)
H1B—C1—H1C	109.5	O1^i^—Ba1—Ba1^iii^	124.48 (6)
Ba1—Cl1—Ba1^i^	88.50 (3)	O1—Ba1—Ba1^iii^	79.47 (5)
Ba1—Cl2—Ba1^ii^	99.32 (2)	Cl1—Ba1—Ba1^iii^	137.642 (7)
Ba1—Cl2—Ba1^iii^	94.21 (2)	Cl2—Ba1—Ba1^iii^	43.606 (18)
Ba1^ii^—Cl2—Ba1^iii^	93.44 (2)	Cl2^iv^—Ba1—Ba1^iii^	102.98 (2)
Ba1^iv^—Cl4—Ba1^ii^	95.60 (3)	Cl2^v^—Ba1—Ba1^iii^	42.91 (2)
Ba1^iv^—Cl4—Ba1	95.60 (3)	Cl4—Ba1—Ba1^iii^	99.24 (3)
Ba1^ii^—Cl4—Ba1	95.60 (3)	Cl3—Ba1—Ba1^iii^	45.933 (9)
O1^i^—Ba1—O1	69.29 (9)	Ba1^i^—Ba1—Ba1^iii^	117.194 (11)
O1^i^—Ba1—Cl1	70.89 (6)	Ba1^v^—Ba1—Ba1^iii^	62.20 (2)
O1—Ba1—Cl1	69.92 (6)	O1^i^—Ba1—Ba1^iv^	118.10 (6)
O1^i^—Ba1—Cl2	83.11 (6)	O1—Ba1—Ba1^iv^	169.45 (5)
O1—Ba1—Cl2	73.36 (6)	Cl1—Ba1—Ba1^iv^	118.845 (16)
Cl1—Ba1—Cl2	140.52 (2)	Cl2—Ba1—Ba1^iv^	99.356 (16)
O1^i^—Ba1—Cl2^iv^	129.87 (6)	Cl2^iv^—Ba1—Ba1^iv^	40.024 (16)
O1—Ba1—Cl2^iv^	142.06 (6)	Cl2^v^—Ba1—Ba1^iv^	98.624 (16)
Cl1—Ba1—Cl2^iv^	85.41 (2)	Cl4—Ba1—Ba1^iv^	42.200 (17)
Cl2—Ba1—Cl2^iv^	133.71 (3)	Cl3—Ba1—Ba1^iv^	44.067 (9)
O1^i^—Ba1—Cl2^v^	142.50 (6)	Ba1^i^—Ba1—Ba1^iv^	151.522 (11)
O1—Ba1—Cl2^v^	73.31 (5)	Ba1^v^—Ba1—Ba1^iv^	58.899 (11)
Cl1—Ba1—Cl2^v^	99.01 (2)	Ba1^iii^—Ba1—Ba1^iv^	90.0
Cl2—Ba1—Cl2^v^	83.71 (2)	Ba1^v^—Cl3—Ba1	88.135 (18)
Cl2^iv^—Ba1—Cl2^v^	83.06 (2)	Ba1^v^—Cl3—Ba1^vi^	91.865 (18)
O1^i^—Ba1—Cl4	79.44 (6)	Ba1—Cl3—Ba1^vi^	180.000 (5)
O1—Ba1—Cl4	139.76 (6)	Ba1^v^—Cl3—Ba1^iii^	91.865 (18)
Cl1—Ba1—Cl4	123.06 (3)	Ba1—Cl3—Ba1^iii^	88.135 (18)
Cl2—Ba1—Cl4	78.438 (19)	Ba1^vi^—Cl3—Ba1^iii^	91.865 (18)
Cl2^iv^—Ba1—Cl4	77.857 (19)	Ba1^v^—Cl3—Ba1^iv^	88.135 (18)
Cl2^v^—Ba1—Cl4	131.44 (3)	Ba1—Cl3—Ba1^iv^	91.865 (18)
O1^i^—Ba1—Cl3	137.07 (5)	Ba1^vi^—Cl3—Ba1^iv^	88.135 (18)
O1—Ba1—Cl3	125.40 (5)	Ba1^iii^—Cl3—Ba1^iv^	180.000 (9)
Cl1—Ba1—Cl3	149.383 (17)	Ba1^v^—Cl3—Ba1^ii^	180.000 (8)
Cl2—Ba1—Cl3	67.244 (16)	Ba1—Cl3—Ba1^ii^	91.865 (18)
Cl2^iv^—Ba1—Cl3	66.799 (16)	Ba1^vi^—Cl3—Ba1^ii^	88.135 (18)
Cl2^v^—Ba1—Cl3	66.32 (2)	Ba1^iii^—Cl3—Ba1^ii^	88.135 (18)
Cl4—Ba1—Cl3	65.13 (3)	Ba1^iv^—Cl3—Ba1^ii^	91.865 (18)

Symmetry codes: (i) *x*−*y*+1/3, −*y*+2/3, −*z*+1/6; (ii) −*x*+*y*, −*x*, *z*; (iii) *y*, −*x*+*y*, −*z*; (iv) −*y*, *x*−*y*, *z*; (v) *x*−*y*, *x*, −*z*; (vi) −*x*, −*y*, −*z*.

## References

[bb1] Allen, F. H. (2002). *Acta Cryst.* B**58**, 380–388. 10.1107/s0108768102003890 12037359

[bb2] Arion, V. B., Kravtsov, V. Ch., Goddard, R., Bill, E., Gradinaru, J. I., Gerbeleu, N. V., Levitschi, V., Vezin, H., Simonov, Y. A., Lipkowski, J. & Bel’skii, V. K. (2001). *Inorg. Chim. Acta*, **317**, 133–142.

[bb3] Brandenburg, K. (2006). *DIAMOND* Crystal Impact GbR, Bonn, Germany.

[bb4] Drozdov, A. A., Troyanov, S. I., Pisarevsky, A. P. & Struchkov, Y. T. (1994). *Polyhedron*, **13**, 1445–1452.

[bb5] Fenske, D., Baum, G., Wolkers, H., Schreiner, B., Weller, F. & Dehnicke, K. (1993). *Z. Anorg. Allg. Chem.* **619**, 489–499.

[bb6] Harrowfield, J. M., Richmond, W. R., Skelton, B. W. & White, A. H. (2004). *Eur. J. Inorg. Chem.* pp. 227–230.

[bb7] Pi, C., Wan, L., Liu, W., Pan, Z., Wu, H., Wang, Y., Zheng, W., Weng, L., Chen, Z. & Wu, L. (2009). *Inorg. Chem.* **48**, 2967–2975. 10.1021/ic802157g 19271770

[bb8] Sheldrick, G. M. (2008). *Acta Cryst.* A**64**, 112–122. 10.1107/S0108767307043930 18156677

[bb9] Stoe & Cie (2009). *X-AREA*, *X-RED32* and *X-SHAPE* Stoe & Cie GmBh, Darmstadt, Germany.

[bb10] Westrip, S. P. (2010). *J. Appl. Cryst.* **43**, 920–925.

[bb11] Yang, J., Li, L., Ma, J. F., Liu, Y. Y. & Ma, J. C. (2006). *J. Mol. Struct.* **796**, 41–46.

